# Structure and Functional Properties of Proteins from Different Soybean Varieties as Affected by the 11S/7S Globulin Ratio

**DOI:** 10.3390/foods14050755

**Published:** 2025-02-23

**Authors:** Yichen Hou, Lu Huang, Guangliang Xing, Xiaotian Yuan, Xiaoyan Zhang, Dongqing Dai, Xingxing Yuan, Xin Chen, Chenchen Xue

**Affiliations:** 1 College of Food Science and Engineering, Nanjing University of Finance and Economics, Nanjing 210023, China; houyichen0324@163.com; 2 Institute of Industrial Crops, Jiangsu Academy of Agricultural Sciences, Nanjing 210014, China; yuanxiaotian235@163.com (X.Y.); 20170071@jaas.ac.cn (X.Z.); daidongqing13@163.com (D.D.); 20090049@jaas.ac.cn (X.Y.); cx@jaas.ac.cn (X.C.); 3 School of Biology and Food Engineering, Changshu Institute of Technology, Changshu 215500, China; xinggl@cslg.edu.cn; 4 School of Food and Biological Engineering, Jiangsu University, Zhenjiang 212013, China

**Keywords:** soybean varieties, 11S/7S ratio, subunit composition, protein structure, functional properties

## Abstract

Soybean (*Glycine max* (L.) Merrill) is a key source of plant protein, with 7S and 11S globulins being the primary fractions. This study investigated the protein content, protein composition, and 11S/7S globulin ratios of 411 soybean samples, and then selected six varieties (S1, S2, S3, S4, S5, and S6) for the analysis of the protein structures and functional properties. The results revealed that varieties with low 11S/7S ratios (S1, S2, and S3) exhibited relatively high random coil contents (20.11–22.94%) and lower β-sheet contents (34.17–38.37%), suggesting the presence of more loosely structured proteins. S2 showed good solubility (73.21%) and water-holding capacity (WHC) (2.73 g/g), which can improve product quality and yield. In contrast, varieties with high 11S/7S ratios (S4, S5, and S6) demonstrated more compact protein structures, increased surface hydrophobicity, larger particle sizes, lower absolute zeta potential values, and greater oil-holding capacity (OHC) values (7.58–8.48 g/g). S4, in particular, demonstrated superior emulsification properties, with emulsion activity index (EAI) (4.71 m^2^/g) and emulsion stability index (ESI) (58.73 min), which are widely used in the food industry such as in cake, ice cream, and bread. This study provides valuable information for the selection of soybean varieties with optimal 11S/7S ratios for processing soybean products.

## 1. Introduction

Soybean (*Glycine max* (L.) Merrill) is an annual dicotyledonous plant indigenous to China and has served as a staple in the Chinese diet for millennia [1]. Soybean seeds contain approximately 40% protein, indicating that soybeans are a significant source of plant-based protein. In addition to their high protein content, the amino acid profile of soy protein closely aligns with the essential amino acid requirements of animals [2]. Furthermore, soybean protein is rich in bioactive peptides that provide health-promoting benefits, such as reducing the risk of cardiovascular disease, hypertension, and certain types of cancer [3]. Therefore, soybeans are often referred to as “storehouses of high-quality proteins.” Given their rich nutritional value, soy proteins are integral to food formulations, nutritional supplements, and various food manufacturing processes [4].

According to their sedimentation coefficients, soybean proteins are categorized into 2S, 7S, 11S, and 15S fractions. The 7S (β-conglycinin) and 11S (glycinin) globulins account for approximately 70% of the total soy protein content and are the primary components of soybean storage proteins. The 7S globulin is a trimeric protein with a molecular weight ranging from 150 to 180 kDa and is composed mainly of α′ (~76 kDa), α (~72 kDa), and β (~53 kDa) subunits. It does not possess disulfide bonds and is stabilized by hydrophobic and electrostatic interactions. In contrast, 11S globulin is a hexameric protein with a molecular weight between 300 and 400 kDa and is composed of acidic subunits (~35 kDa) and basic subunits (~20 kDa) associated with a single disulfide bond. The relative proportions of 11S and 7S proteins not only affect nutritional quality but also influence the functional characteristics of soy proteins because of their varying amino acid compositions and structures [5]. 7S globulin is well known for its exceptional emulsifying properties, making it especially suitable for producing soymilk and related products. Its simpler and more flexible subunit structure allows the 7S protein to unfold more readily during processing, enhancing its interactions with water and oil and improving its emulsifying capabilities [6]. Conversely, the 11S globulin features a compact structure stabilized by disulfide bonds, resulting in greater gel strength but lower emulsifying and foaming capacities than those of 7S [7]. This characteristic renders it more appropriate for tofu production and similar applications [8]. Thus, the 11S/7S ratio significantly impacts both the nutritional quality and functional properties of soy proteins.

With the growing global demand for plant proteins, understanding the intrinsic properties of soy proteins has become essential for advancing food processing technologies and developing innovative food applications. For example, research on the protein subunit composition of six soybean varieties has demonstrated that the 11S/7S protein ratio in seeds influences tofu yield and gel hardness [9]. Additionally, Ren et al. successfully produced a plant-based soy yogurt with excellent gel and textural properties by adjusting the ratio of 7S to 11S proteins [10]. As the origin of soybean cultivation, China currently hosts a national crop germplasm bank that preserves over 40,000 soybean varieties. Despite this wealth of germplasm resources, the diversity of protein composition and functional properties remains largely unexplored.

In this study, 411 soybean germplasm samples were collected, including 391 from different regions in China and 20 from outside the country. Protein composition analysis was conducted using sodium dodecyl sulfate-polyacrylamide gel electrophoresis (SDS-PAGE), and six varieties (S1, S2, S3, S4, S5, and S6) were identified on the basis of their maximum and minimum 11S/7S values for further examination. The differences in protein structure were analyzed using Fourier transform infrared spectroscopy, ultraviolet absorption spectroscopy, intrinsic fluorescence spectroscopy, surface hydrophobicity analysis, particle size assessment, and zeta potential measurements. Additionally, the water-holding capacity (WHC), oil-holding capacity (OHC), emulsion stability index (ESI), and emulsifying activity index (EAI) were measured to assess the effects of structural variations on functional properties. This investigation examines the influence of 11S/7S ratio differences among soybean varieties on protein structure and functional properties, providing a scientific basis for soy product processing and supporting the evaluation and application of diverse soybean varieties. The study highlights the critical role of 11S/7S ratios in enhancing protein solubility and emulsification properties, advancing rational utilization of soybean protein, and optimizing plant-based protein foods.

## 2. Materials and Methods

### 2.1. Materials

A total of 411 soybean seed samples were collected from diverse regions, both domestically and internationally, by the Institute of Industrial Crops at the Jiangsu Academy of Agricultural Sciences. Among these varieties, 391 soybean varieties originate from 15 provinces in China, whereas the remaining 20 varieties come from three countries: Brazil, Japan, and the United States. All materials were cultivated at the Liuhe Base of the Jiangsu Academy of Agricultural Sciences (Nanjing, China), located at 32.08° N, 118.40° E. 411 soybean samples were planted in June and harvested in October 2023. A randomized complete block design with three replicates was implemented. Each germplasm accession was planted in three rows, with ten holes per row and three seeds per hole. The experimental design utilized a randomized complete block approach with single-row plots and three replicates. The soybeans were harvested at the R8 stage of seed development, freeze-dried, and stored at −80 °C until use.

1-Anilinonaphthalene-8-sulfonic acid (ANS) and N,N,N,N-tetramethylamine (TEMED) were procured from Sigma-Aldrich Biochemical Technology Co., Ltd. (St. Louis, MO, USA). A protein molecular weight marker (15–130 kDa) was obtained from Sangon Biotech Co. (Shanghai, China). All other chemicals used in this study were of analytical grade.

### 2.2. Extraction of Soybean Protein

Protein extraction was conducted following the methods described by Song et al. [11] and Peñas et al. [12]. Soybean samples were ground to a fine powder and passed through a 0.2 mm sieve for later use. Five grams of soybean flour were measured and mixed with hexane at a solid-to-liquid ratio of 1:3 (*w*/*v*) for defatting and then air-dried at room temperature. The defatted soybean flour was combined with 50 mM Tris-HCl buffer (pH 8.0) at a ratio of 1:15 (*w*/*v*), thoroughly mixed, and shaken for extraction for 60 min. The mixture was then centrifuged at 12,000 rpm at 4 °C for 20 min. The resulting supernatant was dialyzed against Tris-HCl buffer and lyophilized for future use.

### 2.3. Protein Content and Composition Analysis

#### 2.3.1. Soluble Protein Content

The soluble protein content in the soybean was evaluated using the Bradford method, with bovine serum albumin as the standard [13]. The absorbance was measured at 595 nm, and the soluble protein content was quantified (g/100 g) via a bovine serum albumin (BSA) standard curve.

To assess the soluble protein content, 1 g of the sample was transferred to a 50 mL conical flask. Protein extraction was performed using 0.05 mol/L Tris-HCl buffer (pH 8.0) on a shaker at 35–40 °C and 120–150 rpm for 1 h. Subsequent ultrasonic extraction was conducted in a water bath for 10 min. The mixture was then centrifuged at 12,000 rpm for 20 min at 4 °C. The oil layer was discarded, and the supernatant was collected. The supernatant was diluted, and 1 mL was mixed with 5 mL of Coomassie Brilliant Blue G-250 solution. The mixture was allowed to react at room temperature for 5 min, after which the absorbance was measured at 595 nm. A blank control was prepared with distilled water. The soluble protein content (g/100 g) was calculated via a BSA standard curve.

#### 2.3.2. Sodium Dodecyl Sulfate-Polyacrylamide Gel Electrophoresis (SDS-PAGE)

Electrophoresis was carried out using a Mini-PROTEAN^®^ Tetra System (Bio-Rad Laboratories, Hercules, CA, USA) within a discontinuous buffer system, featuring a 10% resolving gel and a 5% stacking gel, with a sample volume of 10 μL. The gel was pre-run at an initial voltage of 60 V for 30 min. Upon the sample entering the resolving gel, the voltage was increased to 120 V until electrophoresis was complete. The gels were stained with Coomassie Brilliant Blue R-250 for 4 h and destained with a deionized aqueous acetic acid solution until the background was clear. The gels were scanned using a Tanon 1600 imaging system and analyzed with Quantity One software (version 4.6.2; Bio-Rad Laboratories, Inc.).

### 2.4. Protein Structure Analysis

#### 2.4.1. Fourier Transform Infrared Spectroscopy (FTIR)

One milligram of the lyophilized protein sample was accurately weighed for analysis. An FTIR spectrometer (Nicolet iS10, Thermo Fisher ScientificTM, Waltham, MA, USA) was used to record a 32-scan interferogram. The wavenumber ranged from 4000 to 400 cm^−1^, utilizing 32 scans. The protein’s secondary structure was analyzed using OMNIC software 9.7 and PeakFit version 4.12.

#### 2.4.2. Measurement of UV Absorption Spectra

The protein sample was dissolved in a 10 mM phosphate-buffered saline (PBS) solution at pH 7.0 and mixed thoroughly to prepare a 2 mg/mL protein solution. The UV absorption spectra of each sample were acquired using a UV-visible spectrophotometer (Purkinje General Instrument Co., Ltd., Beijing, China) within the wavelength range of 230–350 nm.

#### 2.4.3. Surface Hydrophobicity (H_0_)

Surface hydrophobicity was determined following the method of Wang et al. [14] using ANS as a fluorescent probe. The extracted protein sample was dissolved in 10 mM PBS at pH 7.0 to prepare a 2 mg/mL protein solution. Subsequently, 20 μL of 8.0 mM ANS solution in PBS was added to 4 mL of the protein mixture, mixed thoroughly, and incubated in the dark for 20 min. The fluorescence intensity was measured with an excitation wavelength of 390 nm, an emission wavelength of 470 nm, and a slit width of 5 nm (F-7000 fluorescence spectrophotometer, Hitachi High-tech Science Corp., Tokyo, Japan). The slope of the fluorescence intensity versus protein concentration was defined as the surface hydrophobicity index.

#### 2.4.4. Intrinsic Fluorescence Spectrometry

Intrinsic fluorescence spectroscopy is sensitive to alterations in protein tertiary structure and can reflect changes in the polarity of the microenvironment surrounding tryptophan (Trp) residues [15]. A 2 mg/mL protein mixture was prepared with 10 mM PBS at pH 7.0. The intrinsic fluorescence emission was measured from 300 to 400 nm using an F-7000 fluorescence spectrophotometer (Hitachi High-tech Science Corp., Japan) with an excitation wavelength of 280 nm.

#### 2.4.5. Particle Size and Zeta Potential Analysis

The particle size was measured via the method described by Chen et al. [16], with appropriate modifications. The zeta potential was determined following the procedure reported by Tang and Sun [17]. The protein sample was dissolved in 10 mM PBS at pH 7.0 to prepare a 1 mg/mL solution, which was then filtered through a 0.45 μm membrane filter. Measurements were conducted using a nanoparticle size and zeta potential analyzer (Nicomp Z3000, Particle Sizing Systems, Port Richey, FL, USA) with a sample volume of 3 mL at a temperature of 25 °C.

### 2.5. Analysis of Protein Functional Properties

#### 2.5.1. Protein Solubility

The protein content was determined using the Coomassie Brilliant Blue assay. A 4 mg/mL protein solution at pH 7 was prepared. The solution was mixed on an orbital shaker for 10 min. The mixture was then centrifuged at 5000 rpm at room temperature for 15 min, and the supernatant was collected. Protein solubility was calculated using the formula below.

(1) $$\mathrm{Protein}\;\mathrm{solubility}(\%)=\frac{\mathrm{Protein}\;\mathrm{content}\;\mathrm{of}\;\mathrm{the}\;\mathrm{supernatant}}{\mathrm{Total}\;\mathrm{protein}\;\mathrm{content}\;\mathrm{before}\;\mathrm{centrifugation}}\;\times \;100$$

#### 2.5.2. Water-Holding Capacity (WHC) and Oil-Holding Capacity (OHC)

The methods used to measure the WHC and OHC of soy protein are outlined by Beuchat [18]. The WHC and OHC are calculated as follows:

(2) $$\mathrm{WHC}\;(\mathrm{OHC})\;=\;\frac{W2\;-\;W1}{W0}$$

where W0 represents the weight of the protein sample, g; W1 represents the total weight of the centrifuge tube and the sample, g; and W2 represents the total weight of the centrifuge tube and the precipitate, g.

#### 2.5.3. ESI and EAI

The ESI and EAI were determined according to the methods established by Pearce and Kinsella [19], with slight modifications. The formulas for calculating the EAI and ESI are as follows:

(3) $${\mathrm{EAI}\;(m}^{2}g^{-1})\;=\;\frac{2\;\times \;2.303\;\times \;A_{500}\;\times \;n}{c\;\times \;\varphi \;\times \;10^{4}}$$

(4) $$\mathrm{ESI}\;(\min)\;=\;\frac{A_{0}}{A_{0}\;-\;A_{10}}\;\times \;10$$

where A_500_ represents the absorbance of the protein emulsion at 500 nm; n represents the dilution factor; *c* represents the protein concentration in the emulsion; *φ* represents the volume fraction of oil in the emulsion; and A_0_ and A_10_ represent the absorbances of the protein emulsion at 500 nm at 0 and 10 min, respectively.

### 2.6. Statistical Analysis

All the measurements were conducted in triplicate, and the results are presented as the means ± standard deviations (SDs). Significant differences in all experimental data were evaluated using SPSS 22.0 software (IBM, Armonk, NY, USA) through a one-way analysis of variance (ANOVA) and Duncan’s multiple comparison tests (*p* < 0.05). Figures were generated using Origin 2021 software (Origin Lab, Inc., Northampton, MA, USA).

## 3. Results and Discussion

### 3.1. Analysis of the Soluble Protein Content and Protein Composition of 411 Soybean Germplasms

**Figure 1:**
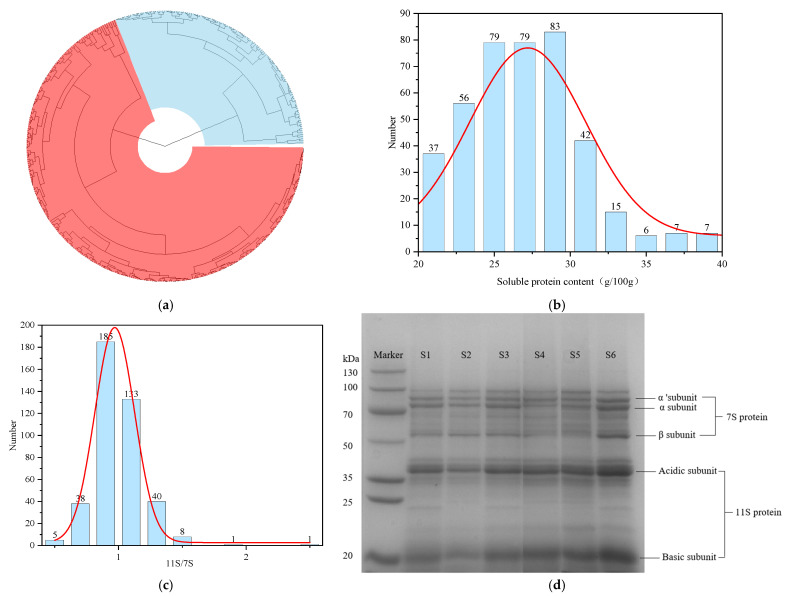
(**a**) Cluster analysis diagram of soybean protein component: red means high varieties with 11S proportion, blue means high varieties with 7S proportion. (**b**) Frequency distribution of soluble proteins content. (**c**) Frequency distribution of 11S/7S ratio. (**d**) The protein profiles of soybean varieties by sodium dodecyl sulfate–polyacrylamide gel electrophoresis.

In this study, we analyzed the soluble protein content and 11S/7S ratios of 411 soybean samples collected from various regions across the country (Table S1). The cluster analysis of 411 soybean germplasm resources, based on 11S and 7S globulin proportions, separated the varieties into two distinct groups: red branches indicating a high 11S proportion and blue branches representing a high 7S proportion. This classification highlights the variability among the soybean varieties (Figure 1a). The soluble protein content ranged from 20 g/100 g to 40 g/100 g, exhibiting a normal distribution, with the highest frequency occurring between 25 g/100 g and 30 g/100 g (Figure 1b). The average soluble protein content was 26.98 g/100 g, with a coefficient of variation (CoV) of 14.25%.

SDS-PAGE is a widely used method for determining the molecular weight distribution of protein subunits (Figure 1d). The gel electropherograms across all the soybean varieties were generally similar, with bands corresponding to the α, α′, β, acidic, and basic subunits. Notably, the widths and intensities of these bands varied among the different soybean varieties, which is consistent with findings from previous studies [20]. The band intensities of the 411 soybean germplasms were quantified using Quantity One, and the 11S/7S ratios were calculated, as illustrated in Figure 1c. The observed 11S/7S ratios ranged from 0.45 to 2.45 and followed a normal distribution, with a CoV of 19%, indicating significant variation among the soybean samples. The 11S/7S seed storage protein ratio is a critical determinant of seeds’ nutritional quality. The 11S protein is of greater nutritional value than the 7S protein due to the high contents of cysteine and methionine. Increasing the 11S/7S ratio directly improves protein quality in both seeds and derived soybean products such as soybean meal. Given the critical role of the 11S/7S ratio in determining the functional and nutritional properties of soybean protein, we selected three samples with the highest 11S/7S ratios and three with the lowest ratios for further examination. These six varieties are designated S1 through S6, where S1–S3 correspond to varieties with low 11S/7S ratios, and S4–S6 represents those with high ratios. Among these, S4 has the highest 11S/7S ratio of 2.45, whereas S3 has the lowest ratio of 0.54 (Table 1). Compared with those of the other varieties, the lighter β subunit bands in S4 and S5 suggest a lower content of 7S proteins, contributing to a higher 11S/7S ratio. Conversely, the relatively lighter basic subunit bands in S1–S3 indicate a diminished content of 11S proteins, resulting in a lower 11S/7S ratio.

The physicochemical properties, structure, and function of soybean proteins are closely associated with the content and ratio of 7S globulins to 11S globulins [21]. The unique amino acid compositions of 11S globulins and 7S globulins confer different properties to these proteins, suggesting that the 11S/7S ratio significantly influences the structural and functional attributes of soybean proteins. At present, some researchers have begun to pay attention to the breeding of soybean varieties with high 11S/7S ratios. Some researchers have developed breeding materials with the α-subunit deletion of 7S globulin which resulted in a high 11S/7S ratio, through artificial mutagenesis or by utilizing natural variation, to meet the needs of specific populations [22]. By concentrating on these six selected varieties, we aimed to elucidate the differences in protein structure and functional properties, thereby providing valuable references for the application of soybean protein.

### 3.2. FTIR Analysis

**Figure 2:**
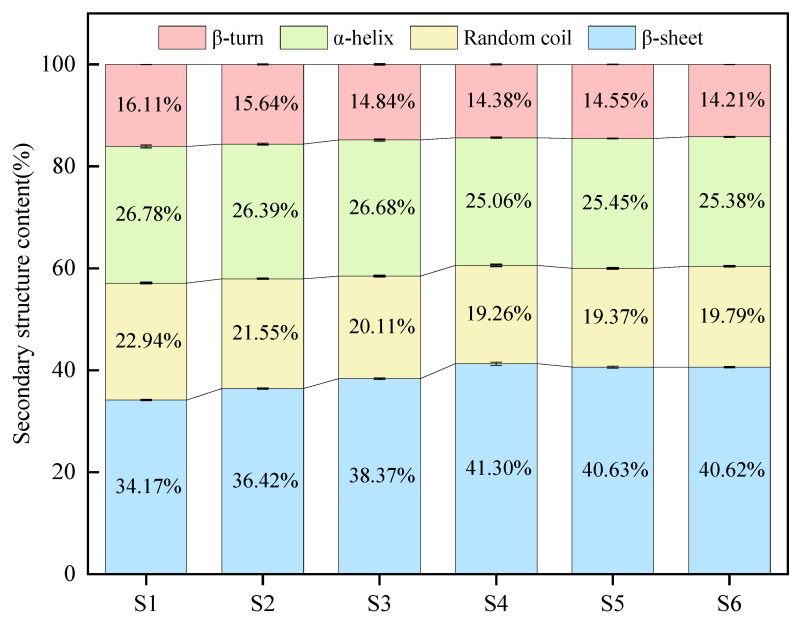
Effect of 11S/7S ratio on protein secondary structures of different soybean varieties.

The secondary structure of soybean varieties with varying 11S/7S ratios was analyzed using FTIR. The FTIR spectra of the proteins predominantly displayed characteristic amide I bands ranging from 1600 to 1700 cm^−1^. Variations in this amide I region indicate changes in the protein’s secondary structure, primarily resulting from the stretching vibrations of the amide C=O bonds [23]. The secondary structures include α-helices (1648–1664 cm^−1^), β-sheets (1615–1637 cm^−1^ and 1682–1700 cm^−1^), β-turns (1664–1681 cm^−1^), and random coils (1637–1648 cm^−1^) [24]. As depicted in Figure 2, the β-sheet content was the highest across all the soybean varieties, followed by the α-helix content, whereas the β-turn content was the lowest. The 11S/7S ratio significantly influences the secondary structure content of soybean proteins. In samples with lower ratios, the β-sheet content ranged from 34.17% to 38.37%, whereas the α-helix content was greater, varying from 26.39% to 26.78%.

β-Sheets consist of parallel or antiparallel chains stabilized by hydrogen bonds, leading to a more compact conformation. The observed lower β-sheet content in samples with lower 11S/7S ratios suggests a reduction in the number of hydrogen bonds between peptide chains. Conversely, the increased prevalence of random coils (20.11–22.94%) implies that these proteins may possess a more relaxed secondary structure, increasing their susceptibility to structural unfolding [25]. A diminished α-helix content has been associated with increased flexibility in protein structures [26]. This structural flexibility is vital for the functional properties of many proteins, including enhanced emulsification capabilities. Consequently, soybean varieties exhibiting higher 11S/7S ratios, which have lower α-helix contents, may demonstrate improved emulsification capacity, particularly in sample S4 [27].

### 3.3. Intrinsic Fluorescence Spectrometry Analysis

**Figure 3:**
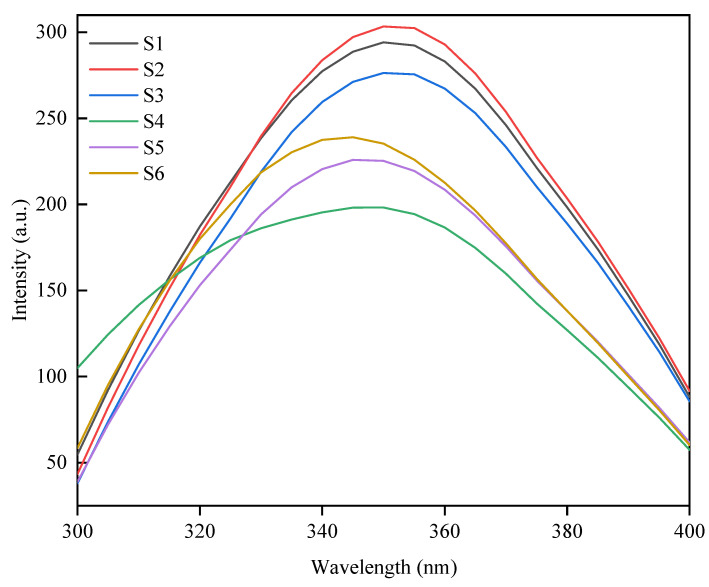
Intrinsic fluorescence spectroscopy of proteins from different soybean varieties.

The intrinsic fluorescence spectra of proteins are influenced by the chemical environments surrounding fluorescent amino acids. Trp typically has a maximum emission wavelength between 330 and 360 nm, whereas tyrosine (Tyr) falls within the 303–310 nm range, and phenylalanine (Phe) ranges between 280 and 285 nm. The fluorescence characteristics of these amino acids are significantly affected by their microenvironments. Variations in environmental polarity result in shifts in chromophore spectra, which correlate with changes in protein structure, particularly the tertiary structure [28]. As illustrated in Figure 3, the maximum emission wavelengths of all samples were concentrated at approximately 350 nm, suggesting that the spectral features are predominantly influenced by Trp. The samples with low 11S/7S ratios showed higher fluorescence intensity values. This may be attributed to the large amount of Trp residues exposed in the environment, resulting in the presence of high fluorescence intensity. The samples with low 11S/7S ratios displayed maximum emission wavelengths ranging from 351.07 to 357.07 nm, whereas those with higher ratios ranged from 347.96 to 350 nm. Notably, a redshift was observed in samples with low 11S/7S ratios, indicating a more open protein conformation and a hydrophilic microenvironment surrounding the Trp residues. Monitoring the differences in fluorescence spectra among these soybean varieties offers a deeper understanding of the microenvironmental changes occurring at each amino acid residue and their interactions within the protein structure. A redshift typically signifies an extended protein conformation and increased polarity within the Trp environment [29].

### 3.4. UV Absorption Spectra Analysis

**Figure 4:**
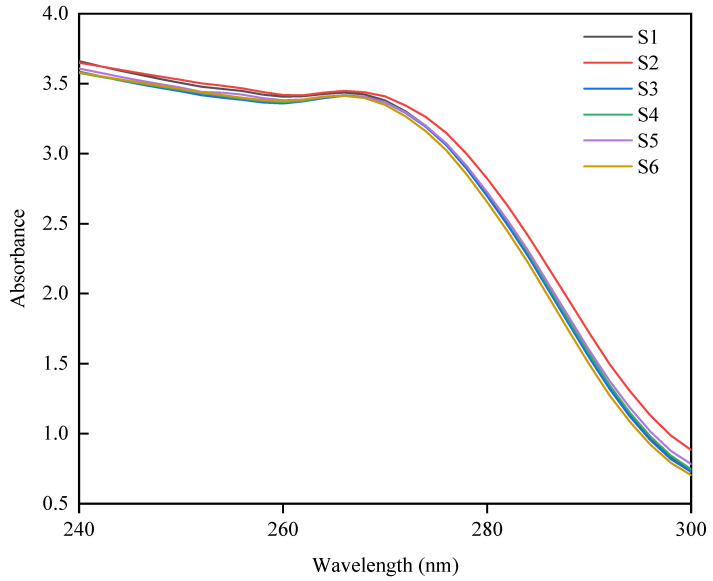
Ultraviolet absorption spectra of proteins from different soybean varieties.

The UV absorption spectra are primarily associated with the absorption characteristics of the Tyr and Trp residues. An increase in UV absorption intensity generally indicates that Tyr and Trp residues are more accessible to the solvent [30]. Consequently, variations in protein conformation can be reflected in the UV absorption spectra. Most proteins exhibit characteristic absorption peaks within the wavelength range of 250–280 nm. Structural differences in proteins can be identified by comparing shifts in these absorption peaks and variations in absorbance. As depicted in Figure 4, samples with lower 11S/7S ratios demonstrate a maximum absorption wavelength at 266 nm, whereas those with higher ratios peak at 264 nm. Furthermore, samples with lower 11S/7S ratios have greater UV absorption intensities, suggesting that more aromatic amino acid residues are exposed on the protein surface. This increased exposure implies a more polar and hydrophilic microenvironment, which corroborates the findings in Figure 3, further indicating that samples with lower ratios possess more open protein conformations.

### 3.5. Surface Hydrophobicity (H_0_)

**Figure 5:**
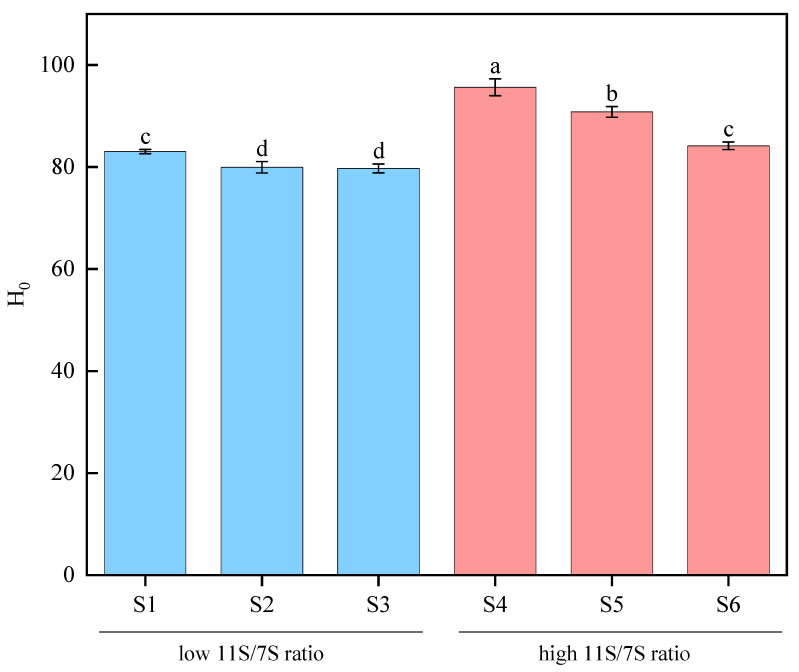
Surface hydrophobicity of proteins from different soybean varieties. Value with different letters is significantly different (*p* < 0.05).

Surface hydrophobicity serves as a vital indicator for detecting conformational changes in proteins, reflecting their propensity to aggregate or dissolve. Moreover, surface hydrophobicity (H_0_) influences functional properties such as solubility and emulsification [31]. As shown in Figure 5, the H_0_ values of the various samples significantly varied, ranging from 79.72 to 95.64. The soybean varieties with lower 11S/7S ratios presented lower H_0_ values. This observation may be attributed to the looser protein structures in samples with low 11S/7S ratios, which promote the exposure of hydrophilic groups, thereby decreasing the H_0_ value. Research indicates that H_0_ is positively correlated with the β-sheet content and negatively correlated with the α-helix content in protein structures [26], a trend that is consistent with the FTIR data presented in Figure 2. Elevated H_0_ values signify a greater presence of nonpolar hydrophobic groups on the protein surface, thereby enhancing the protein’s capacity to interact with hydrophobic molecules or surfaces and offering additional binding sites for fluorescent probes [32]. Furthermore, studies have shown that the solubility of proteins from different soybean varieties is negatively correlated with surface hydrophobicity, as more soluble proteins tend to have fewer hydrophobic residues on their surfaces [33].

### 3.6. Zeta Potential and Particle Size

**Figure 6:**
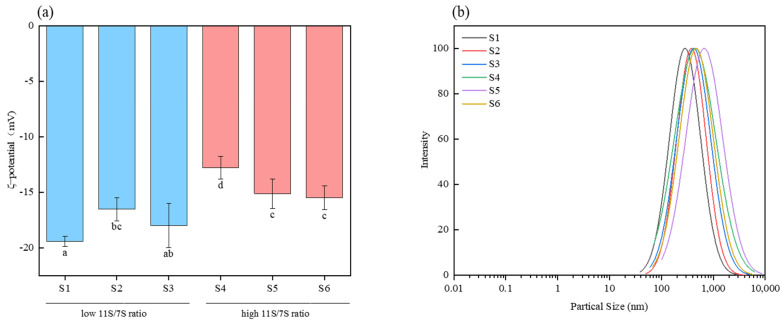
(**a**) ζ-potential of proteins from different soybean varieties. (**b**) Particle size distribution of soy protein from different varieties. Value with different letters is significantly different (*p* < 0.05).

Proteins function as amphoteric electrolytes, possessing either negative or positive charges depending on the pH of their environment [34]. As depicted in Figure 6a, all samples exhibited negative zeta potential values, attributed to their presence in a buffer solution with a pH of 7.0, which exceeds the isoelectric point of soy protein (approximately 4.5). Under these conditions, soy protein has a negative charge. The zeta potential characterizes the electrostatic interactions between particles and serves as a key indicator of system stability [35]. A greater absolute value of the zeta potential indicates increased system stability. Zeta potential primarily reflects the colloidal stability of proteins, enhancing their stability in beverages, preventing sedimentation and phase separation, and finally extending the product’s shelf life. Figure 6a shows that soybean proteins with low 11S/7S ratios presented higher absolute zeta potential values (16.54–19.41) than those with high 11S/7S ratios (12.79–15.49). Consequently, solutions containing proteins with low 11S/7S ratios were more stable than those with high 11S/7S ratios. Additionally, at pH 7.0, emulsions utilizing 7S globulin as the emulsifier demonstrated higher absolute zeta potential values than those employing 11S globulin, suggesting that 7S globulin surfaces carry a greater charge [36]. This finding corroborates our conclusions.

As illustrated in Figure 6b, the particle sizes of the soy proteins predominantly ranged from 100 to 1000 nm, with all the samples demonstrating a single-peak particle size distribution. Notably, soybean varieties exhibiting higher 11S/7S ratios typically presented larger particle sizes than those with lower ratios. This observation implies that soy proteins with elevated 11S/7S ratios are more likely to form protein aggregates. These aggregates could arise from inter- or intramolecular noncovalent interactions, such as hydrophobic interactions, which are closely associated with increased surface hydrophobicity [37]. Moreover, a correlation exists between protein particle size and zeta potential: smaller particle sizes are associated with enhanced protein solubility in solution and greater resistance to aggregation, thereby improving system stability, an association evident in higher absolute values of zeta potential. This conclusion aligns with our findings. Notably, the subunit composition of soybean varieties impacts their particle size distribution. An increase in the acidic subunits A1 and A2 within the 11S fraction may facilitate the formation of larger proteins, which could explain the slightly larger particle sizes observed in proteins with higher 11S/7S ratios [38].

### 3.7. Protein Solubility

**Figure 7:**
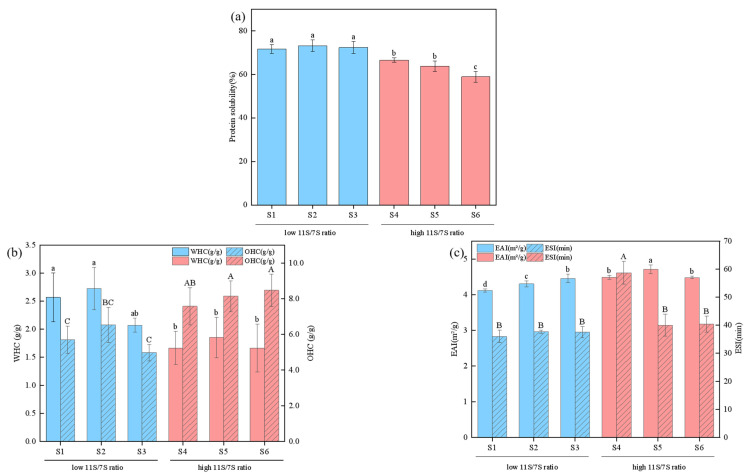
Effect of 11S/7S ratio on functional properties of different soybean varieties. (**a**) Protein solubility, (**b**) WHC and OHC, (**c**) ESI and EAI. Value with different letters is significantly different (*p* < 0.05).

Solubility is a critical functional property of proteins and affects other attributes, such as emulsification and gelation [39]. As depicted in Figure 7a, the protein solubility among different soybean varieties varied from 58.93% to 73.21%, with significant differences noted between samples (*p* < 0.05). Proteins characterized by low 11S/7S ratios presented an average solubility of 72.42%, which was higher than the 63.1% observed in samples with high 11S/7S ratios, suggesting that lower 11S/7S ratios are conducive to higher solubility. This difference may be attributed to the reduced surface hydrophobicity (Figure 5) and enhanced solution stability (Figure 6a). The soybean variety S2 demonstrated the highest solubility at 73.21%. Proteins with high solubility are widely utilized as raw materials in various functional foods, including sports beverages, protein supplements, and whey protein products. Consequently, proteins with lower 11S/7S ratios are better positioned for these applications.

The composition of 11S globulins, 7S globulins, and their respective subunits significantly influences protein solubility. Owing to the more rigid structure of its fundamental polypeptide chains, 11S globulin is less soluble than 7S globulin. Consequently, the content of 11S is negatively correlated with protein solubility [40], which is consistent with the results of our experiments. Furthermore, the solubility of 7S globulin is affected by the makeup of its α′, α, and β subunits. Although the core regions of these subunits are highly hydrophobic, the extended regions of the α′ and α subunits contain a greater concentration of polar amino acid residues, rendering them more soluble than the β subunit [41].

### 3.8. WHC and OHC

The functional properties of soy proteins, such as WHC and OHC, play crucial roles in their processing and application within food systems. As depicted in Figure 7b, significant differences (*p* < 0.05) in WHC were observed across different soybean varieties. Varieties with lower 11S/7S ratios presented slightly higher WHC values (2.07–2.72 g/g) than those with higher ratios (1.67–1.85 g/g). This phenomenon may be attributed to the effects of amino acid composition, protein spatial conformation, and the balance between surface polarity and hydrophobicity on the WHC [42]. The soybean variety S2, which has a high WHC, may be particularly suitable for producing food products that require moisture absorption and retention, such as bakery products.

In terms of OHCs, proteins with elevated 11S/7S ratios presented higher OHC values, ranging from 7.58 to 8.48 g/g. The highest OHC value of 8.48 g/g was noted in sample S6, whereas the lowest value of 4.97 g/g was observed in S3. The OHC value is indicative of the hydrophobicity of soybean protein, with proteins exhibiting greater surface hydrophobicity being more prone to interact with fats and oils, as hydrophobic regions tend to bind with nonpolar substances. Hence, soy proteins with greater hydrophobicity also present increased OHCs, which aligns with our findings [43]. Among the six evaluated varieties, sample S6 presented the highest OHC value, making it a suitable candidate for use as a plant-based meat substitute. Currently, soy protein is recognized as one of the most essential ingredients in plant-based meat alternatives [44]. Varieties exhibiting higher OHCs are particularly well suited for food products that necessitate fat retention, such as meat and dairy products, owing to their superior fat absorption capabilities.

In addition, the 11S/7S ratio also influences other functional properties, such as foaming properties and gelation, which are crucial in the food industry. Higher 11S/7S ratios typically lead to reduced foaming ability and enhanced gelation [45,46].

### 3.9. ESI and EAI

The EAI measures a protein’s ability to quickly form a stable layer at the oil-water interface, whereas the ESI evaluates its capacity to stabilize emulsions and prevent the dissociation of the adsorbed layer [47]. The EAI and ESI values of different varieties of soybean proteins significantly vary. As illustrated in Figure 7c, the EAI of soy protein ranged from 4.12 to 4.71 m^2^·g^−1^, and the ESI values ranged from 36.06 to 58.73 min. Compared with those with a lower ratio, the soybean varieties with a higher 11S/7S ratio presented slightly elevated EAI and ESI values. Specifically, S4 had the highest EAI and ESI, measuring 4.49 m^2^/g and 58.73 min, respectively. This may be attributed to partial protein unfolding, which exposes additional hydrophobic amino acid residues and increases surface hydrophobicity. Consequently, this increase in surface activity and adsorption at the oil-water interface is notable [48].

Emulsifiability is a critical functional property of proteins and is widely utilized in the food industry in products such as salad dressings, mayonnaise, and frozen desserts. This property not only enhances nutritional value but also improves texture and mouthfeel [49]. These results indicate that the soybean variety S4 has excellent emulsifiability, making it a suitable candidate for the targeted production of soy protein isolates with emulsifying properties. The different 11S/7S ratios of soybean varieties have a significant impact on the functional properties of proteins, so the screening and rational utilization of high-quality varieties are important for the processing of soy-based products.

## 4. Conclusions

This study investigated the protein content and composition of 411 soybean germplasms and six special materials with different 11S/7S ratios were selected for the protein structure and functional properties analysis. The results showed that varieties with a high 11S/7S ratio exhibited more compact protein structures, higher surface hydrophobicity, improved emulsifying properties, and higher OHC. In contrast, varieties with a low 11S/7S ratio demonstrated looser protein structures, higher WHC, and protein solubility. This study demonstrates that different 11S/7S ratios of soybean germplasms have a significant impact on the structure and function of soybean protein, which in turn affects processing quality. The 11S/7S ratio can also be used as an indicator of the nutritional quality of soybean protein. The above findings not only provide excellent germplasms to produce soy-based products but also provide important data for breeders in the selection and breeding of functional soybean varieties. In the future, more attention should be paid to the entire soybean industry chain, from resource screening and variety breeding to germplasm utilization and product processing.

## Figures and Tables

**Table 1:** Soluble protein content, protein composition, and 11S/7S ratio of selected six soybean varieties.

Sample	Genotype Name	Soluble Protein Content (g/100 g)	Source/Origin	7S (%)			11S (%)		11S/7S
				α′ Subunit	α Subunit	β Subunit	Acidic Subunit	Basic Subunit	
S1	Jiangningxiaoyadou	28.39 ± 0.91 a	Jiangsu province (East of China)	9.10	9.45	13.84	7.59	11.58	0.59
S2	Shangpei	25.57 ± 0.93 b	Jiangsu province (East of China)	8.72	8.44	13.25	6.53	10.27	0.55
S3	Rudong	28.10 ± 1.05 a	Jiangsu province (East of China)	8.75	9.09	12.48	7.10	9.36	0.54
S4	Fengxiansunlou	24.48 ± 0.38 bc	Jiangsu province (East of China)	9.15	7.94	0.00	18.88	22.92	2.45
S5	Blackjack	22.21 ± 0.85 d	The United States	6.70	9.05	7.24	14.23	19.27	1.46
S6	Qihuang34	23.78 ± 1.00 c	Shandong province (North of China)	4.39	8.17	7.88	14.63	17.46	1.57

Value with different letters is significantly different (*p* < 0.05).

## Data Availability

The original contributions presented in this study are included in the article/Supplementary Material. Further inquiries can be directed to the corresponding authors.

## Supplementary Materials

The following supporting information can be downloaded at https://www.mdpi.com/article/10.3390/foods14050755/s1. Table S1: Soluble protein content, protein composition, and 11S/7S values of 411 soybean germplasm resources from different regions.
