# Supplementary material for: Structure and Functional Properties of Proteins from Different Soybean Varieties as Affected by the 11S/7S Globulin Ratio
Source: Foods. 2025 Feb 23;14(5):755. doi: 10.3390/foods14050755 (PMC11899569; doi:10.3390/foods14050755)
Supplement: Supplementary file 1 [file foods-14-00755-s001.zip › foods-3438963-supplementary.pdf]

Supporting Information

Table S1. Soluble protein content, protein composition, and 11S/7S values of 411 soybean germplasm resources from different regions

Sample	Soluble protein content(g/100g)	α' subunit (%)	α subunit (%)	β subunit (%)	Acidic subunit (%)	Basic subunit (%)	11/7S
1	21.31±0.67	7.56	9.16	10.13	12.51	15.14	1.03
2	20.71±0.17	9.59	10.51	8.61	13.62	15.01	1.00
3	23.26±0.25	8.15	7.73	10.43	13.77	15.54	1.11
4	34.01±1.29	10.34	8.33	13.59	8.24	13.12	0.66
5	28.53±1.22	9.53	9.99	14.35	8.27	12.65	0.62
6	25.15±1.33	8.37	10.25	9.87	12.72	14.83	0.97
7	24.90±0.70	8.66	10.06	8.03	19.26	14.02	1.24
8	21.51±0.47	8.88	11.78	9.31	18.39	14.75	1.11
9	22.91±0.16	9.54	11.62	8.81	18.16	13.33	1.05
10	21.51±0.30	9.77	7.75	8.73	20.84	14.87	1.36
11	23.36±0.43	8.70	7.53	8.35	21.49	15.64	1.51
12	24.82±0.12	9.40	11.46	7.33	21.30	13.70	1.24
13	27.97±2.17	8.67	9.63	9.63	13.24	13.38	0.95
14	22.78±0.22	9.33	11.53	7.35	19.91	12.95	1.16
15	23.66±0.86	7.82	8.60	9.08	12.82	13.71	1.04
16	20.99±0.41	9.97	8.34	8.83	19.39	14.17	1.24
17	24.48±0.38	8.50	7.38	0.00	17.55	21.30	2.45
18	21.39±0.20	7.98	8.27	9.32	13.08	13.54	1.04
19	23.64±0.19	7.38	6.16	8.12	22.23	17.10	1.82

20	22.41±0.36	8.92	8.45	8.62	13.72	13.91	1.06
21	25.38±0.53	12.06	12.16	11.51	14.91	16.81	0.89
22	25.14±0.73	11.65	11.97	11.06	13.54	15.23	0.83
23	24.80±0.23	12.34	12.28	11.61	15.20	17.34	0.90
24	21.48±0.96	13.13	13.44	10.61	14.94	16.41	0.84
25	24.55±1.00	13.28	12.41	11.49	15.38	17.18	0.88
26	20.43±1.26	8.20	9.43	10.05	13.36	13.55	0.97
27	22.06±0.51	6.22	6.78	11.01	11.37	15.14	1.10
28	24.68±0.69	12.06	12.53	10.69	14.30	14.95	0.83
29	21.24±0.57	9.14	8.52	9.31	13.75	14.02	1.03
30	22.13±0.69	12.71	12.68	11.71	14.62	16.15	0.83
31	22.66±0.60	12.80	12.16	11.77	14.31	15.32	0.81
32	22.53±0.68	11.59	11.21	11.27	12.90	13.64	0.78
33	22.73±0.86	9.08	8.50	10.46	13.66	14.25	1.00
34	21.66±1.06	8.21	7.49	9.34	14.51	16.27	1.23
35	20.88±2.49	8.51	8.10	8.70	13.66	14.73	1.12
36	24.38±0.60	8.42	7.96	9.78	13.49	14.35	1.06
37	31.02±0.39	7.78	8.84	8.22	13.60	15.05	1.15
38	28.34±1.29	7.92	7.74	9.99	14.05	14.51	1.11
39	28.35±1.03	7.87	6.61	8.77	15.79	11.45	1.17
40	27.44±0.79	7.60	8.63	7.18	16.22	11.11	1.17
41	30.01±0.36	8.08	6.94	9.24	16.39	11.19	1.14
42	32.19±0.36	7.75	9.32	9.03	14.23	14.70	1.11
43	21.45±0.49	7.59	7.36	10.13	13.91	14.69	1.14
44	28.80±0.86	6.83	8.06	10.47	12.69	14.58	1.08
45	26.57±1.02	7.94	9.07	11.15	12.84	13.62	0.94

46	23.93±2.39	8.20	9.29	9.52	12.82	14.42	1.01
47	24.99±0.84	7.90	9.25	10.62	13.20	14.62	1.00
48	29.33±0.13	8.60	8.14	10.30	13.07	15.47	1.06
49	20.61±1.44	8.63	9.50	7.98	11.86	12.43	0.93
50	26.56±0.91	8.62	9.46	7.75	11.85	12.27	0.93
51	25.15±0.45	8.84	9.56	9.09	9.77	10.91	0.75
52	22.32±0.53	8.30	9.15	8.06	11.47	12.01	0.92
53	25.26±1.18	8.02	8.74	7.69	11.63	12.74	1.00
54	26.13±2.19	8.26	9.20	6.39	11.25	12.45	0.99
55	22.17±0.84	9.90	8.78	10.55	12.41	14.71	0.93
56	27.56±0.58	9.09	10.64	10.73	13.10	14.24	0.90
57	25.00±0.11	9.26	10.75	9.88	13.29	14.80	0.94
58	26.65±0.11	9.74	10.01	10.64	12.96	14.24	0.90
59	21.58±0.47	9.15	10.66	9.38	14.04	15.92	1.03
60	23.31±0.45	9.00	10.58	10.47	13.20	15.13	0.94
61	24.28±0.19	9.14	10.75	11.22	13.06	14.65	0.89
62	23.69±0.10	8.54	9.78	10.39	13.56	15.50	1.01
63	25.07±0.37	9.28	10.67	10.04	14.99	17.05	1.07
64	28.06±0.44	8.44	9.67	9.74	15.35	17.35	1.17
65	32.05±0.37	7.68	8.83	12.49	15.14	17.23	1.12
66	28.12±0.37	8.66	7.52	10.46	15.35	18.61	1.27
67	28.63±0.37	8.18	9.87	10.94	14.48	17.30	1.10
68	27.35±0.58	8.42	7.63	11.57	15.71	18.52	1.24
69	24.04±0.49	8.28	9.87	9.67	14.38	18.42	1.18
70	25.35±0.36	5.62	0.00	10.91	13.99	21.89	2.17
71	31.89±0.51	8.98	10.51	9.43	14.73	16.81	1.09

72	32.89±0.29	7.99	8.33	11.59	13.52	15.60	1.04
73	27.32±0.20	8.50	9.62	9.24	13.93	16.80	1.12
74	26.09±0.67	9.67	10.57	10.73	12.34	12.96	0.82
75	30.33±0.67	9.18	9.87	11.16	12.30	13.47	0.85
76	28.92±0.83	9.24	10.37	10.83	12.21	13.49	0.84
77	21.59±0.26	9.94	11.19	11.09	13.43	14.35	0.86
78	28.22±0.19	7.72	9.03	9.93	13.89	14.30	1.06
79	29.64±0.38	7.66	8.17	10.87	13.80	14.37	1.06
80	27.77±0.62	8.27	9.49	8.23	15.02	12.49	1.06
81	22.24±0.40	9.68	10.63	8.88	13.53	15.31	0.99
82	21.37±1.62	9.49	10.87	9.17	12.64	13.73	0.89
83	24.50±1.28	10.03	11.17	9.60	12.47	13.92	0.86
84	22.81±2.16	8.81	9.47	7.88	13.92	15.53	1.13
85	30.61±0.71	8.37	8.72	8.43	14.34	16.91	1.22
86	27.10±0.45	9.34	9.90	10.46	14.11	17.52	1.06
87	21.08±0.35	9.68	10.06	8.51	13.19	14.31	0.97
88	26.01±0.69	9.61	11.44	10.59	13.93	15.42	0.93
89	24.33±1.21	9.62	9.61	10.65	15.42	16.33	1.06
90	25.93±0.17	7.81	8.89	9.71	14.54	17.64	1.22
91	22.08±1.11	9.55	11.47	8.01	15.89	16.84	1.13
92	21.08±0.69	9.02	11.10	11.35	14.88	16.10	0.98
93	22.02±1.07	9.33	11.48	8.31	14.68	16.16	1.06
94	28.04±0.90	8.72	10.70	10.23	14.12	15.44	1.00
95	24.41±0.55	8.79	9.92	11.34	13.48	14.93	0.95
96	24.61±0.15	7.90	8.79	11.12	14.54	18.09	1.17
97	28.58±0.25	8.19	9.74	11.33	16.08	17.85	1.16

98	38.21±0.39	5.97	9.48	11.48	14.06	17.88	1.19
99	28.59±1.38	9.67	10.36	8.28	12.77	13.39	0.92
100	33.30±0.25	8.97	10.66	10.99	14.34	16.58	1.01
101	24.24±0.41	9.82	10.39	8.73	13.38	14.65	0.97
102	22.02±0.27	9.27	10.46	8.69	13.51	15.53	1.02
103	20.30±0.68	7.29	8.61	10.29	14.95	19.46	1.31
104	26.32±0.47	7.26	8.62	10.75	14.76	19.09	1.27
105	22.51±0.17	9.72	10.26	8.40	13.84	15.05	1.02
106	24.71±0.26	7.05	8.55	9.57	14.36	20.16	1.37
107	24.14±0.23	7.08	7.42	12.03	13.36	20.06	1.26
108	27.99±0.76	8.90	9.13	9.27	12.69	13.54	0.96
109	20.06±0.26	9.71	8.20	8.37	14.36	16.32	1.17
110	27.22±0.95	9.23	9.96	9.91	12.79	13.57	0.91
111	24.38±0.18	6.54	7.14	9.35	14.35	21.65	1.56
112	27.20±0.50	8.59	8.80	10.70	14.65	18.93	1.20
113	32.81±0.63	10.12	11.11	8.42	13.95	15.71	1.00
114	26.70±1.27	10.47	11.71	7.76	14.23	16.25	1.02
115	26.86±0.60	9.95	11.33	10.14	14.63	16.36	0.99
116	39.94±0.14	10.07	11.24	11.48	13.65	14.50	0.86
117	24.99±0.64	7.49	8.27	10.81	15.24	21.37	1.38
118	24.09±0.26	7.89	8.12	9.31	14.68	21.61	1.43
119	24.34±0.14	7.87	8.47	11.97	14.10	19.28	1.18
120	39.97±0.59	9.47	10.18	10.18	13.90	15.81	1.00
121	26.66±1.02	9.00	9.66	8.34	13.42	14.38	1.03
122	39.39±0.12	8.38	10.02	10.84	13.40	15.83	1.00
123	28.01±1.29	8.63	8.99	7.41	12.46	16.23	1.15

124	28.66±1.15	8.51	8.33	8.81	13.06	16.19	1.14
125	30.13±1.03	9.35	10.12	8.09	14.02	15.56	1.07
126	27.48±1.02	8.65	8.23	9.20	10.93	14.35	0.97
127	23.14±0.77	8.18	9.15	11.44	15.11	19.34	1.20
128	26.51±1.32	8.79	8.85	9.18	13.09	14.11	1.01
129	25.06±0.26	8.32	9.22	11.58	14.95	21.21	1.24
130	24.21±0.74	8.39	9.16	10.71	14.38	20.81	1.25
131	25.59±0.96	8.27	9.11	10.27	15.46	20.61	1.30
132	23.70±1.50	8.37	9.04	8.94	15.96	20.56	1.39
133	27.07±0.44	8.60	10.00	10.11	12.46	13.56	0.91
134	23.39±0.44	8.36	9.56	9.37	11.94	12.76	0.91
135	28.11±0.68	9.51	9.68	8.13	14.80	16.67	1.15
136	22.82±0.35	8.95	9.95	10.37	12.23	13.14	0.87
137	21.80±0.25	8.66	9.70	9.83	11.66	12.88	0.87
138	25.18±0.24	8.63	9.75	9.56	11.81	12.49	0.87
139	22.82±0.69	8.58	9.39	9.93	11.52	12.09	0.85
140	21.80±0.09	9.98	10.57	9.93	10.80	10.71	0.71
141	25.18±0.39	9.60	10.08	9.56	10.23	10.97	0.73
142	26.84±0.72	9.46	10.21	9.40	12.53	12.65	0.87
143	25.77±0.92	8.49	9.74	9.41	13.04	14.95	1.01
144	21.11±0.34	7.82	9.16	13.09	16.03	19.40	1.18
145	24.23±0.24	8.21	9.53	12.50	16.15	19.28	1.17
146	26.46±0.40	8.42	9.99	10.90	16.17	18.76	1.19
147	27.84±1.59	8.77	10.08	7.23	14.61	18.19	1.26
148	26.23±0.32	8.89	10.38	10.87	16.23	18.21	1.14
149	28.99±0.68	8.99	9.96	7.37	15.48	19.05	1.31

150	27.95±0.76	8.36	9.57	13.08	15.26	18.48	1.09
151	28.01±0.89	8.21	9.25	9.28	13.39	16.02	1.10
152	28.73±1.13	8.90	9.82	7.92	14.12	16.07	1.13
153	27.91±1.12	8.40	9.57	10.37	12.54	14.54	0.96
154	20.57±0.29	8.59	9.83	10.27	11.34	13.47	0.86
155	22.25±0.48	8.05	10.03	13.47	16.93	18.76	1.13
156	26.57±1.21	8.78	9.96	7.85	12.36	13.96	0.99
157	28.61±1.15	8.79	9.49	7.78	12.65	14.61	1.05
158	28.75±1.48	7.77	7.39	11.76	6.75	10.18	0.63
159	28.39±0.91	8.07	8.38	12.27	9.68	10.27	0.69
160	26.23±0.32	6.23	6.79	13.20	16.04	20.61	1.40
161	27.95±0.44	7.59	8.92	12.35	17.03	19.16	1.25
162	31.84±0.79	8.24	9.42	9.06	14.29	15.16	1.10
163	26.90±0.90	7.97	9.47	9.59	13.77	15.16	1.07
164	25.57±0.93	7.80	7.55	11.85	8.69	9.19	0.66
165	28.37±0.84	7.03	8.89	9.88	14.33	16.13	1.18
166	26.10±1.04	7.42	8.56	10.96	13.70	15.15	1.07
167	26.62±0.43	7.89	9.46	8.77	14.16	15.39	1.13
168	28.71±0.93	8.30	10.22	9.32	14.34	14.61	1.04
169	28.13±1.58	7.76	9.64	10.76	13.74	14.66	1.01
170	26.51±1.70	7.61	8.31	11.28	13.36	15.14	1.05
171	27.90±1.49	7.61	8.94	10.97	14.30	15.61	1.09
172	28.15±0.43	7.95	9.09	10.60	13.59	15.76	1.06
173	30.31±0.76	7.34	9.00	9.74	13.65	15.39	1.11
174	25.93±1.67	8.39	10.17	11.87	12.99	13.68	0.88
175	28.10±0.30	7.51	9.39	8.62	14.26	15.57	1.17

176	24.22±1.27	7.62	9.68	10.56	13.24	14.10	0.98
177	30.77±1.01	7.40	7.95	11.92	13.21	14.57	1.02
178	26.44±1.83	8.26	10.03	11.94	13.01	14.25	0.90
179	24.33±0.87	7.95	9.64	11.09	14.13	15.35	1.03
180	28.70±1.56	8.01	10.53	9.98	13.72	15.86	1.04
181	28.33±0.37	7.18	8.78	11.07	12.45	13.94	0.98
182	26.82±0.17	8.48	9.72	7.56	13.54	17.32	1.20
183	26.51±0.34	6.52	7.92	9.15	12.83	14.94	1.18
184	30.42±0.96	7.06	8.66	8.29	12.49	14.96	1.14
185	39.20±0.81	8.45	9.95	9.16	14.03	17.49	1.14
186	28.66±1.80	6.95	8.75	8.41	12.87	15.51	1.18
187	28.10±1.05	7.92	8.23	11.30	6.43	8.48	0.54
188	28.70±0.43	8.35	9.06	9.17	13.94	17.67	1.19
189	31.82±0.71	6.60	9.12	11.49	12.48	17.90	1.12
190	38.11±0.73	8.53	9.47	5.93	11.72	12.39	1.01
191	37.69±0.35	7.93	8.85	9.24	14.83	17.97	1.26
192	29.66±0.83	7.01	8.89	10.37	12.83	14.73	1.05
193	28.99±0.68	8.27	9.68	7.60	15.35	17.67	1.29
194	24.90±0.23	7.78	9.44	9.13	13.86	17.27	1.18
195	30.31±1.21	5.88	8.24	9.36	13.76	17.02	1.31
196	29.30±1.36	6.62	8.57	8.92	12.57	17.14	1.23
197	29.04±1.34	8.14	8.11	10.71	6.17	9.14	0.57
198	32.86±1.51	7.00	8.72	10.15	13.47	15.59	1.12
199	28.30±1.23	9.92	11.77	14.06	18.28	19.21	1.05
200	27.24±1.01	9.43	10.85	12.19	13.54	14.71	0.87
201	31.37±0.30	8.43	9.58	9.92	13.75	16.68	1.09

202	27.48±0.39	8.79	10.22	10.47	12.78	13.85	0.90
203	27.22±1.18	8.94	9.26	13.23	5.81	8.49	0.45
204	29.62±0.25	8.92	10.08	9.93	12.59	14.52	0.94
205	25.71±1.25	8.37	10.07	11.31	12.56	14.41	0.91
206	30.97±0.74	9.30	10.59	10.86	12.79	14.91	0.90
207	23.55±0.17	8.89	10.84	11.26	12.72	13.90	0.86
208	28.10±0.79	8.59	10.06	10.24	13.59	14.72	0.98
209	27.61±1.38	9.53	10.66	10.03	12.76	13.85	0.88
210	27.79±2.20	9.00	10.64	10.06	15.98	16.39	1.09
211	31.08±1.06	9.34	8.29	10.37	12.65	13.57	0.94
212	24.42±0.60	7.88	9.31	11.04	14.45	14.70	1.03
213	25.30±1.78	9.18	10.35	8.12	12.45	14.22	0.96
214	28.99±0.68	8.91	10.47	9.80	12.13	13.13	0.87
215	24.90±0.23	9.05	10.83	9.84	12.57	13.58	0.88
216	28.79±0.66	8.46	10.19	11.92	13.57	13.61	0.89
217	23.35±1.33	8.45	8.92	8.73	11.92	13.62	0.98
218	27.48±0.39	8.40	10.55	8.80	12.35	14.34	0.96
219	29.44±0.56	9.08	11.09	8.52	12.90	13.99	0.94
220	25.59±0.19	8.42	9.86	11.89	13.40	12.97	0.87
221	25.71±1.25	8.38	10.40	9.20	13.26	14.43	0.99
222	36.05±2.10	9.35	10.60	12.03	15.44	15.03	0.95
223	31.17±0.26	9.37	11.05	11.02	16.48	16.13	1.04
224	30.97±0.74	9.24	10.72	9.51	13.15	14.30	0.93
225	24.48±0.19	8.06	8.73	7.45	12.20	12.65	1.03
226	25.24±0.42	10.79	11.54	13.82	15.74	15.59	0.87
227	23.55±0.17	9.06	10.92	10.71	14.23	15.29	0.96

228	28.10±0.79	9.18	10.49	9.61	12.69	13.65	0.90
229	27.61±1.38	9.24	10.82	9.95	11.56	12.38	0.80
230	31.08±1.06	9.97	9.61	9.86	12.78	13.10	0.88
231	35.51±0.57	10.17	11.10	10.55	14.18	14.40	0.90
232	27.41±1.51	9.01	10.22	10.52	12.80	13.50	0.88
233	25.48±1.47	9.55	10.50	8.05	12.72	13.37	0.93
234	24.81±0.56	9.50	9.12	9.63	14.73	16.63	1.11
235	21.21±1.11	7.55	7.64	9.59	13.88	16.28	1.22
236	31.11±0.36	9.31	10.17	11.65	14.48	15.57	0.97
237	35.99±0.18	9.75	10.81	8.67	14.13	14.71	0.99
238	36.15±1.37	9.49	10.33	8.59	14.63	15.06	1.05
239	36.70±0.31	9.15	10.59	8.93	14.39	15.07	1.03
240	33.33±0.66	9.58	10.94	8.60	16.33	17.62	1.17
241	38.68±0.25	10.02	10.86	9.09	14.49	17.34	1.06
242	36.24±0.71	9.60	11.18	6.40	16.73	24.65	1.52
243	31.61±0.64	7.86	9.62	4.76	17.73	27.05	2.01
244	35.06±0.72	11.70	12.88	11.34	16.34	17.85	0.95
245	24.28±1.64	8.70	7.15	6.75	14.68	17.05	1.40
246	23.74±1.43	7.90	9.21	7.70	14.85	15.46	1.22
247	28.40±1.12	9.02	10.08	6.69	14.06	14.53	1.11
248	21.57±0.97	8.77	8.93	5.55	13.50	15.51	1.25
249	23.12±1.15	9.22	9.66	7.13	14.10	15.27	1.13
250	21.86±0.31	8.94	9.40	5.35	14.32	15.11	1.24
251	27.36±3.14	8.78	9.52	8.01	14.89	15.65	1.16
252	26.00±1.21	9.02	9.03	7.79	15.11	16.02	1.20
253	23.26±0.64	9.89	10.19	4.87	12.97	15.41	1.14

254	25.59±1.12	8.29	8.98	9.91	12.91	13.80	0.98
255	23.83±0.35	7.89	8.73	8.39	12.81	14.05	1.07
256	26.23±0.43	8.14	9.15	7.94	13.84	14.92	1.14
257	31.62±1.30	8.14	9.05	7.34	12.70	13.81	1.08
258	25.42±0.88	7.79	8.24	8.52	12.15	13.84	1.06
259	23.32±0.89	7.89	8.97	7.39	12.12	13.43	1.05
260	29.96±0.67	8.41	8.61	9.00	11.64	12.70	0.94
261	31.69±1.20	8.43	8.83	8.97	11.86	12.85	0.94
262	24.97±0.60	8.45	8.54	7.40	10.10	12.72	0.94
263	29.63±0.94	8.62	9.03	8.19	14.17	17.07	1.21
264	21.86±0.37	8.97	9.78	6.78	12.94	15.39	1.11
265	26.93±0.24	9.73	10.00	6.49	13.81	15.31	1.11
266	28.31±1.22	7.93	7.49	9.26	13.65	16.09	1.21
267	25.96±0.71	8.29	8.80	5.26	13.02	16.04	1.30
268	22.88±0.75	8.63	9.31	8.30	14.23	15.60	1.14
269	24.79±0.97	8.24	9.75	8.33	13.57	16.18	1.13
270	26.51±0.94	7.84	8.50	5.52	13.37	16.66	1.37
271	30.67±0.86	8.28	9.65	8.88	13.52	14.98	1.06
272	27.69±0.82	8.47	9.11	7.25	10.74	13.62	0.98
273	30.48±0.90	8.66	9.87	8.50	10.73	12.09	0.84
274	31.83±0.59	8.66	9.30	9.30	10.58	13.21	0.87
275	29.15±1.10	8.77	9.73	7.34	11.40	14.22	0.99
276	24.77±1.22	8.24	9.31	8.45	10.95	14.11	0.96
277	24.27±1.98	8.77	9.17	7.87	11.36	13.74	0.97
278	23.93±0.52	8.70	9.70	9.81	11.51	13.71	0.89
279	21.49±0.69	8.44	9.05	7.97	10.31	15.20	1.00

280	25.15±0.79	8.24	9.07	8.88	10.27	14.76	0.96
281	26.78±1.61	8.74	9.14	7.77	9.62	10.69	0.79
282	24.50±1.13	9.37	9.78	7.61	10.11	11.37	0.80
283	27.62±0.59	8.98	9.47	7.84	10.12	10.82	0.80
284	25.64±1.32	8.65	9.03	8.58	9.83	10.53	0.78
285	23.99±0.92	8.67	8.85	8.46	9.72	10.79	0.79
286	24.15±0.76	8.44	8.67	7.30	9.65	11.91	0.88
287	25.08±0.11	8.34	8.65	7.38	9.49	11.62	0.87
288	26.92±0.79	8.55	8.61	8.14	9.95	10.68	0.82
289	25.15±1.57	8.73	9.12	9.01	10.17	11.58	0.81
290	26.51±2.58	8.25	7.67	8.82	10.42	12.92	0.94
291	23.19±0.40	8.42	8.64	7.59	10.72	13.34	0.98
292	22.68±0.23	7.95	8.20	6.96	10.13	14.31	1.06
293	22.91±1.32	8.70	8.83	7.59	9.92	13.62	0.94
294	32.15±1.05	8.76	8.99	7.20	10.59	13.66	0.97
295	31.76±1.14	9.58	9.87	9.68	10.56	10.40	0.72
296	28.32±1.55	8.73	9.36	9.15	10.23	13.00	0.85
297	28.20±0.51	8.14	8.19	8.39	10.16	13.80	0.97
298	29.69±0.37	8.70	9.00	7.09	10.87	13.78	0.99
299	30.23±1.54	8.25	8.85	7.92	10.46	13.14	0.94
300	32.75±1.74	8.71	8.81	8.32	10.59	13.07	0.92
301	29.07±1.50	8.88	9.58	8.74	10.79	13.39	0.89
302	26.80±1.46	8.56	9.30	8.90	10.31	13.95	0.91
303	26.45±1.52	9.25	9.84	9.52	9.82	14.43	0.85
304	27.69±1.49	9.11	9.64	9.03	11.37	14.56	0.93
305	21.14±0.94	9.29	10.32	8.54	11.07	11.57	0.80

306	28.03±0.64	8.39	7.66	9.03	9.97	9.97	0.80
307	31.74±0.49	9.12	10.15	9.21	10.77	10.38	0.74
308	22.67±0.89	9.54	10.46	8.87	10.86	10.61	0.74
309	26.38±0.56	8.77	9.14	8.79	10.48	9.97	0.77
310	26.39±0.44	8.86	9.64	7.25	10.55	10.46	0.82
311	23.26±0.80	8.74	9.80	8.84	10.38	10.21	0.75
312	25.80±1.28	8.98	9.58	8.84	10.26	9.97	0.74
313	25.57±0.45	8.56	8.90	8.84	10.02	9.83	0.75
314	20.94±1.52	7.51	7.23	9.38	9.48	10.48	0.83
315	23.56±0.51	8.06	9.01	7.86	9.81	10.27	0.81
316	20.51±1.20	8.86	9.67	8.33	10.39	10.12	0.76
317	23.75±0.96	8.23	0.00	10.70	10.89	10.80	1.15
318	23.57±1.40	8.88	9.44	8.61	10.09	9.95	0.74
319	20.50±0.73	9.27	9.94	8.78	10.78	10.36	0.76
320	20.03±1.45	9.28	9.91	6.41	10.89	10.76	0.85
321	27.31±2.16	8.89	9.47	8.67	10.11	10.12	0.75
322	22.15±0.87	9.05	9.42	8.18	10.31	10.42	0.78
323	28.39±0.67	8.63	9.02	8.70	9.54	9.53	0.72
324	26.33±0.92	7.79	7.28	8.72	9.06	9.14	0.77
325	22.14±2.02	9.45	10.40	8.98	10.75	10.93	0.75
326	30.29±0.83	8.69	9.24	8.09	9.68	9.82	0.75
327	25.02±0.49	8.12	0.00	9.63	10.04	10.25	1.14
328	26.93±1.13	8.34	9.11	7.88	9.64	9.78	0.77
329	28.16±0.15	8.14	8.64	6.86	9.04	9.07	0.77
330	27.95±0.08	7.97	8.40	7.98	9.67	10.07	0.81
331	29.87±0.94	7.93	8.51	7.54	9.35	9.47	0.78

332	28.15±0.21	8.54	9.50	8.75	9.93	9.85	0.74
333	25.99±4.79	8.46	9.20	7.46	10.03	10.27	0.81
334	30.37±0.65	7.83	8.93	9.57	10.06	10.29	0.77
335	35.74±0.60	8.62	9.05	8.66	9.44	9.38	0.71
336	32.07±0.42	8.93	9.49	10.50	9.34	9.68	0.66
337	30.49±0.34	8.23	8.84	7.47	9.78	10.01	0.81
338	29.74±0.54	7.81	7.17	10.30	10.43	11.22	0.86
339	26.93±1.06	8.43	9.83	8.23	9.97	10.99	0.79
340	31.78±0.50	9.11	9.79	7.03	10.16	10.44	0.79
341	30.70±0.31	8.65	9.30	7.44	11.53	12.15	0.93
342	29.48±0.92	9.14	9.66	10.06	12.37	12.74	0.87
343	27.45±1.36	9.41	9.35	7.42	11.91	11.61	0.90
344	21.65±0.73	8.39	9.19	7.70	11.53	12.49	0.95
345	29.13±0.71	8.51	9.33	8.57	11.44	12.25	0.90
346	27.93±0.71	9.65	10.49	8.42	12.08	12.24	0.85
347	28.83±0.44	8.19	8.95	7.58	11.33	11.50	0.92
348	28.56±0.73	9.16	9.88	8.58	11.60	11.57	0.84
349	28.01±0.83	10.03	10.52	7.61	12.58	12.74	0.90
350	31.24±0.88	8.73	9.41	9.88	11.67	12.33	0.86
351	33.65±0.50	9.01	9.96	8.18	11.38	11.78	0.85
352	33.62±0.83	8.97	9.45	5.95	11.70	12.17	0.98
353	31.01±0.58	7.35	8.86	7.21	11.83	13.68	1.09
354	26.34±1.05	7.17	9.05	7.49	11.37	13.87	1.06
355	28.42±0.4	7.25	6.99	9.97	11.68	13.60	1.04
356	28.23±1.51	7.49	9.66	8.96	11.59	13.50	0.96
357	28.06±0.48	7.04	8.62	8.35	10.78	13.39	1.01

358	28.61±0.41	7.64	9.71	9.60	12.10	13.95	0.97
359	30.93±0.93	6.19	5.76	11.02	10.85	13.22	1.05
360	30.13±0.82	6.74	9.65	8.63	10.38	12.92	0.93
361	32.37±0.36	7.63	9.48	10.90	10.40	13.28	0.85
362	28.38±0.83	8.36	9.84	9.12	12.18	12.32	0.90
363	28.34±1.55	8.23	10.10	10.46	11.67	13.40	0.87
364	28.10±1.45	7.52	9.15	8.90	11.60	13.73	0.99
365	28.98±1.18	8.84	8.95	8.39	12.28	12.76	0.96
366	27.38±1.99	8.49	8.77	8.81	11.77	13.37	0.96
367	28.99±2.05	8.78	9.43	9.09	12.06	12.94	0.92
368	25.19±0.84	8.51	0.00	11.40	11.68	12.23	1.20
369	29.28±0.63	8.94	9.40	9.02	10.54	10.51	0.77
370	24.92±1.17	8.16	7.07	9.00	11.12	11.58	0.94
371	24.39±0.58	9.01	9.41	8.45	11.59	11.81	0.87
372	23.42±0.47	9.18	9.85	8.38	11.22	11.57	0.83
373	28.16±4.42	9.04	8.74	8.20	12.21	12.86	0.96
374	30.41±0.33	7.94	7.92	8.68	10.09	10.62	0.84
375	32.29±0.83	9.43	10.04	7.97	11.70	11.77	0.86
376	29.77±0.76	8.46	8.89	8.65	10.04	10.48	0.79
377	30.63±0.75	8.44	8.73	8.89	12.46	13.99	1.01
378	37.11±0.68	9.13	9.30	9.37	12.64	12.83	0.92
379	31.34±1.64	8.57	8.68	9.21	11.48	12.16	0.89
380	31.33±0.70	8.62	9.87	8.61	11.50	11.79	0.86
381	28.36±1.43	8.47	9.16	0.00	11.99	13.19	1.43
382	29.19±1.01	8.95	9.36	9.02	13.30	13.72	0.99
383	30.83±0.75	8.33	8.52	10.26	11.48	11.98	0.87

384	32.29±0.57	9.02	10.00	10.26	11.52	12.36	0.82
385	21.87±0.47	8.19	9.10	9.50	11.83	14.29	0.97
386	36.49±0.61	8.78	9.33	8.31	11.71	12.49	0.92
387	25.81±0.21	8.57	9.85	8.96	11.96	12.60	0.90
388	27.41±0.70	8.96	9.46	9.11	12.12	12.22	0.88
389	28.31±0.47	9.41	10.02	8.62	12.92	13.43	0.94
390	24.99±0.57	8.32	10.31	9.26	13.41	13.75	0.97
391	28.00±1.10	9.01	9.60	8.78	12.43	12.69	0.92
392	27.77±0.85	9.12	9.49	5.99	11.92	13.14	1.02
393	27.05±2.46	8.69	10.09	8.80	11.42	13.63	0.91
394	26.81±0.63	8.81	9.79	7.27	11.48	12.84	0.94
395	23.46±0.99	9.73	10.35	6.98	14.03	14.18	1.04
396	24.05±0.74	8.17	9.79	8.33	17.42	17.16	1.32
397	22.21±0.85	6.36	8.59	6.88	13.51	18.30	1.46
398	21.75±0.63	8.80	9.09	6.94	15.01	15.46	1.23
399	24.35±1.66	9.51	10.64	6.42	18.03	17.65	1.34
400	29.53±0.21	8.93	10.26	10.31	13.51	13.62	0.92
401	25.19±1.60	8.23	9.55	8.27	14.16	14.92	1.12
402	26.23±1.01	9.65	9.29	7.51	14.85	14.87	1.12
403	29.38±1.09	9.32	10.03	8.15	12.20	11.82	0.87
404	26.64±1.23	9.23	9.81	7.44	12.93	12.52	0.96
405	24.96±0.44	9.36	9.61	8.67	11.03	11.90	0.83
406	22.59±0.32	9.51	9.70	8.10	11.18	11.24	0.82
407	23.87±0.59	8.78	8.66	9.55	10.99	11.34	0.83
408	27.34±0.31	8.67	8.94	8.49	12.51	12.77	0.97
409	34.66±0.71	8.53	9.44	8.80	11.87	11.50	0.87

410	23.78±1.00	4.39	8.17	7.88	14.63	17.46	1.57
411	29.68±1.51	9.05	9.60	7.44	12.52	12.74	0.97
